# Massive open online nutrition and cooking course for improved eating behaviors and meal composition

**DOI:** 10.1186/s12966-015-0305-2

**Published:** 2015-12-03

**Authors:** Maya Adam, Kelly C. Young-Wolff, Ellen Konar, Marilyn Winkleby

**Affiliations:** Department of Pediatrics, Stanford School of Medicine, Stanford University, Building 20, Main Quad, Stanford, CA 94305 USA; Department of Medicine, Stanford Prevention Research Center, Stanford, CA USA; Stanford Center for Advanced Studies in the Behavioral Sciences, Stanford, CA USA

**Keywords:** Nutrition, Cooking, Online education, Eating behaviors, Meal composition

## Abstract

**Background:**

Behavioral shifts in eating, favoring the increased consumption of highly processed foods over healthier, home-cooked alternatives, have led to widespread health problems. This study reports on the effectiveness of a massive open online course (MOOC), offering integrated nutrition and cooking instruction, for improving eating behaviors and meal composition among course participants.

**Methods:**

The course, consisting of 47 short (4–6 min.) videos, was offered through Coursera, an open, online learning platform, available to individuals worldwide who have access to the Internet. Beginning in January 2014, participants viewed course videos, completed quizzes and participated in optional cooking assignments, over a 5-week period. Participants were invited to complete optional pre- and post-course surveys assessing their eating behaviors, typical meal composition and perceived barriers to home cooking. McNemar-Bowker tests of symmetry and within subject *t*-tests were conducted to evaluate pre-post survey changes in the primary variables measured.

**Results:**

7,422 participants from more than 80 countries completed both pre- and post-course surveys, while 19,374 participants completed the pre-survey only. Class participants were primarily women in the child-rearing ages (20–49 years of age). There were significant positive changes in eating behaviors and meal composition over time, including an increase in the percentage of participants who reported cooking dinner at home using mostly fresh ingredients 5–7 times in the previous week (63.4 % to 71.4 %), and who felt that yesterday’s dinner was very/extremely healthy (39.3 % to 56.4 %) and enjoyable (55.2 % to 66.7 %) (all *p* values < .0001).

**Conclusions:**

Integrated nutrition and cooking courses, delivered via open online learning platforms, offer a free and flexible venue for reaching adults worldwide and have the potential to catalyze powerful behavioral shifts that align well with efforts to improve eating behaviors and meal composition.

## Background

The harmful effects of over-consumption of highly processed food have been well-documented [1–3]. Access to traditional, didactic nutrition education has been shown to elicit positive changes in eating behaviors among adults, particularly when nutrition education is explicitly linked to disease prevention [4–6]. The protective influence of healthful home cooking and the family meal on child and adolescent health outcomes, including obesity prevention, are also well documented [7–11]. This has led to calls for culinary skills education for school children, parents and healthcare providers [9, 11, 12]. Prior research testing the hypothesis that “the inclusion of ‘culinary education’ (e.g., cooking demonstrations and hands-on cooking) as adjuncts to traditional didactic, nutrition presentations would result in measurable positive changes in both personal and professional nutrition-related behaviors” has yielded encouraging results among adults [12]. Similarly, in adolescents, both cooking education and family meals have been found to increase fruit and vegetable intake [13–15] among other positive changes in eating behavior and meal composition.

Parents have been identified as key players in establishing and modeling long-term eating behaviors for their children [6], but many parents model sub-optimal eating behaviors themselves [16] and identify barriers to both home cooking and the acquisition of nutrition education. Especially in the US, increasing the average intake of fruits and vegetables has been identified as a priority for health promotion and disease prevention efforts [17–20]. In addition, avoidance of highly processed foods, eating family meals and cooking at home have all been identified as health-promoting behaviors [2, 8, 13, 21] that lead to improved dietary intake in various populations. Major barriers to home cooking that have been identified, include perceived inconvenience [9], time constraints, lack of child care, transportation and low motivation to attending traditional nutrition education classes [6].

Online courses present an opportunity to overcome many logistical barriers to access for both traditional didactic nutrition education classes and culinary skills classes. While past, fee-based, online nutrition courses, have been found to enhance knowledge, student satisfaction has been mixed and the need to address emerging technologies has been identified [5]. The emergence of free, online learning platforms presents an opportunity to meet the needs of a diverse, worldwide audience. Since both lower socioeconomic status and level of education are known risk factors for poor dietary intake [22], minimizing barriers for these groups is particularly important.

Despite the need for healthful eating behaviors among adults and children, and the growing accessibility of massive open online learning [23], there are limited free online nutrition courses produced by medical and nutrition science professionals for the primary purpose of improving family eating behaviors and meal composition. Additionally, no prior massive open online courses (MOOCs) have integrated basic online cooking education and online didactic nutrition education. To address this important gap, a medical and nutrition science team at the Stanford School of Medicine created and evaluated a MOOC aimed at promoting widespread access to cooking education and integrated didactic nutrition education. The objective of this pilot study was to assess the effectiveness of open, online nutrition and cooking instruction in improving the eating behaviors of course participants.

## Methods

### Course development and curriculum

A pilot version of the Stanford Child Nutrition and Cooking MOOC was offered on Coursera in May 2013. The course was primarily intended to reach participants with children, but individuals without children could participate as well. As with all Coursera courses, participants could choose to watch videos multiple times and the number of views per video was tracked by Coursera. More than 20,000 students enrolled in the 5-week introductory-level course and 60 % of enrollees engaged with course material beyond enrollment. More than half of these active participants provided feedback on the course, via discussion forums and/or course surveys. Feedback from the pilot course was used to generate a list of the participants’ most frequently cited “topics of interest”. Guided by the Social Cognitive Theory [24, 25] of behavior change, the course developers used this list to develop the Stanford Child Nutrition and Cooking 2.0 MOOC, offered from early January to mid-February 2014. This 5-week course was offered in English and consisted of 47 short (4–6 min.), easy-to-understand, instructional videos. Integrated instructional cooking videos aimed to provide participants with the tools to increase their skills and self-efficacy for healthful cooking by providing consistent on-screen modeling. In line with the Social Cognitive Theory of behavior change, course instructors, a medical doctor, a nutrition scientist and a chef, modeled healthful cooking, family mealtimes and involving children in the cooking process. The consistent integration of explanation, demonstration and modeling was intended to facilitate observational learning among course participants, while improving self-efficacy. Furthermore, the course employed a solution-oriented approach [26] by focusing largely on the potential benefits of healthful home cooking, rather than the risks associated with poor food choices, with the goal of demonstrating more immediate relevance to course participants.

### Eligibility

The Stanford Child Nutrition and Cooking 2.0 MOOC was a free, open-access course available to individuals worldwide who had access to the internet. Both the pilot version and the final version of the course were advertised to Coursera’s existing student population and through promotional materials released by Stanford’s Vice Provost for Online Learning. According to Coursera’s Terms of Use, individuals under the age of 13 are excluded from enrollment. Participants were expected to spend 2–4 h per week engaging with the course content and assignments.

### Course content

The course content videos comprised three categories:

- *Category 1*: “Basic Concept” videos covered paradigm-level concepts supporting healthy eating behaviors, including sensible grocery shopping, moderation in the diet, (including, for example, moderating intake of red meat), benefits of the family meal and how to encourage children to try unfamiliar foods.
- *Category 2*: “Focus Point” videos described basic nutrition education concepts including introductions to macronutrients, micronutrients, US nutrition guidelines, the physiology of taste and other related concepts.
- *Category 3*: “How To” videos consisted of short cooking demonstrations, interspersed with nutrition information about the foods being prepared. These videos delivered basic culinary skills education with an emphasis on preparing healthy meals economically, in a time-efficient manner.

Across all three categories of content, five health messages, each identified as priorities in past nutrition research, were emphasized throughout the course: 1) health benefits of cooking at home, using mostly fresh ingredients, including adequate fresh fruits and vegetables [27], 2) health and psychosocial benefits of eating family meals [21], 3) health benefits of reducing processed food consumption [28, 29], 4) health and environmental benefits of eating sustainably grown foods [30, 31], and 5) health benefits of practicing dietary moderation [32, 33].

The course content was then organized into a self-paced, 5-week curriculum, with a 10–15 question quiz at the end of each week. Participants were allowed to repeat quizzes, in line with literature suggesting that formative testing of this nature reduces student anxiety and solidifies learning [34]. In addition, participants were given the opportunity to communicate with each other and with teaching staff via online discussion forums and social media platforms. Coursera granted a Statement of Accomplishment to all participants who achieved a final grade of 60 % or higher and “distinction” was granted to all participants who achieved a grade of 80 % or higher on the weekly course quizzes. Optional weekly cooking assignments were not included in the calculation of the final grade, but participants were encouraged to share photographs and recipes for their home cooked meals with members of the class community. During the course, feedback was obtained informally via the discussion forums that contributed to ongoing adjustments in the logistics of course delivery. Before and after the course, participants were asked to complete a confidential online survey that assessed their socio-demographic background, cooking and eating behaviors, and perceived barriers to home cooking.

The Stanford IRB granted a Human Subjects waiver as this project was defined as a program evaluation rather than a systematic investigation.

### Measures

#### Demographic variables

Demographic information was assessed in the pre-survey and included gender, age, education, race/ethnicity, country of residence, living situation (live alone, with family, with friends/other, with child younger than 18) and baseline perceived weight status.

#### Eating behaviors and meal composition

Eating behaviors and meal composition were assessed at both pre-and post-surveys for both the past week and for the previous day’s dinner. Participants indicated the number of days over the *past week* that: 1) they ate dinner at home (all food types), 2) their oldest child under the age of 18 ate dinner at home with a family member, and 3) they cooked at home using mostly fresh foods; responses were categorized as follows: 0 days = 0, 1–4 days = 1, 5–7 days =2. Participants also reported on the previous day’s dinner, including whether it was cooked at home using mostly fresh foods (no = 0, yes = 1), whether it included fresh vegetables, fresh fruit, poultry, fish, red meat, vegetable protein, dairy, starch, and baked goods/sweets (no = 0, yes = 1, for each food type), as well as their levels of enjoyment and perceived healthfulness of the previous night’s dinner rated on five-point Likert scales and categorized as follows: not enjoyable or somewhat enjoyable (coded 0), moderately enjoyable (coded 1), and very or extremely enjoyable (coded 2); not healthy or somewhat healthy (coded 0), moderately healthy (coded 1) and very or extremely healthy (coded 2).

### Perceived ease of making healthy food choices and home cooked meals

Perceived ease or difficulty of making healthy food choices and preparing home cooked meals, a measure of self-efficacy, was assessed using eight questions, including how easy or difficult is it to avoid processed foods, get fresh foods to cook healthy meals, decide what to cook, cook using fresh foods, eat the right amount of food, get time to cook a meal at home, cook a meal that you enjoy, and eat healthy. Responses were gathered using a five-point Likert scale and categorized difficult or very difficult (coded 0), neutral (coded 1), and easy or very easy (coded 2); Cronbach’s alpha = 0.80. An overall index score was also created using the summed total scores from these eight items.

### Analysis

Survey responses were collected through Qualtrics, an online survey software platform, and all analyses were computed using SAS statistical software version 9.13^1^. Participants were able to skip survey questions and, for each analysis, we included only participants who responded to each particular question at both the pre- and post-surveys. Thus, the Ns across different questions vary slightly. McNemar-Bowker tests of symmetry were conducted to test for pre- to post-survey differences in categorical variables, including eating behaviors, meal composition, and changes in perceived ease or difficulty of making healthy food choices and preparing home cooked meals. Finally, the study authors tested for pre- to post-survey changes in the three primary outcomes (consumption of fresh vegetables at yesterday’s dinner, consumption of fresh fruits at yesterday’s dinner, and total perceived ease of healthy cooking) by key demographic characteristics, including gender, age, education, baseline perceived weight, and US residence vs. non-US residence, to see whether changes would only occur for certain demographic groups. Stratified McNemar-Bowker tests of symmetry were used to test pre- to post-survey changes in consumption of fresh vegetables and fruits (categorical variables) and paired *t*-test analyses were used to test pre- to post-survey changes in total perceived ease of healthy cooking (continuous variable).

## Results

In line with published data on other MOOCs, a large discrepancy between number of enrolled students and number of course participants was seen [35]. Of the 35,521 students enrolled in the Stanford Child Nutrition and Cooking course, 22,023 were course participants who engaged with any course material beyond enrollment. The pre-course survey was completed by 19,374 (88.0 % of participants), over a 7-day period preceding the release of the first course videos. Both pre- and post-course surveys were completed by 7,422 (33.7 % of participants). All participants who completed both the pre- and post-surveys were included in the current study. Attrition analyses showed that participants who completed both the pre- and post-surveys were generally similar to those who only completed the pre-survey with respect to demographic characteristics, including education and perceived weight, although they were somewhat younger (79 % vs. 72.6 % under age 40) and more likely to be male (15 % vs. 13 %). Further, there were no differences between those who completed both the pre- and post-surveys and those who only completed the pre-survey on average perceived ease of healthy cooking at baseline (27.3, SD = 5.7 vs. 26.4, SD = 5.9), although they were somewhat more likely to report consuming fresh fruit (28.4 % vs. 25.3 %) and fresh vegetables (71.4 % vs 66.3 %) at yesterday’s dinner at baseline. The overall completion rate for the course, defined as the percentage of enrolled students who received a Statement of Accomplishment (SOA) from Coursera, was 30 % of enrolled students.

The majority of the 7,422 participants were female (86.4 %), white (59.8 %), in their child-bearing and child-rearing years (87.1 % aged 20–49), living with family (86.6 %), including at least one child under age 18 (52.7 %), with at least a 4 year college degree (71.8 %) (Table 1). The course reached participants from more than 80 countries and more than half of the class participants (59.1 %) lived outside of the US. Among those who lived outside of the US, the 5 most common countries of residence were Canada (12 %), India (6 %), the United Kingdom (6 %), Australia (5 %), and Spain (5 %). Approximately 1/3 of participants perceived themselves to be overweight or obese (32.8 %).

**Table 1:** Demographic variables (*N* = 7,422)

	*n*	%
Gender		
Female	6382	86.4
Male	979	13.3
Other	24	0.3
Age Group		
<20	235	3.2
20–29	2087	28.3
30–39	3036	41.1
40–49	1306	17.7
≥50	722	9.8
Education		
< High School	123	1.7
High School degree	370	5.0
Some college/2-year degree	1596	21.6
4 year degree	2741	37.1
Graduate school	2565	34.7
Race/Ethnicity^a^		
African/African American	348	7.5
Asian	1602	21.6
Latino	1000	13.5
White	4437	59.8
Other	513	6.9
Country of Residence		
US	2992	40.9
Outside of US	4329	59.1
Living Situation^a^		
Alone	595	8.0
Family	6424	86.6
Friends/Other	892	7.6
Live with child < 18 years old	3915	52.7
Live with child > 18 years old	498	6.7
Baseline Perceived Weight Status		
Underweight	316	4.3
Neither under nor overweight	4553	61.6
Overweight	2047	27.7
Obese	331	4.5
Unsure	139	1.9

Note. Ns do not always add up to 7,422 due to missing responses

^a^Participants could identify with more than one category

Eating behaviors and meal composition are described in Table 2. Almost all pre/post survey comparisons showed significant changes in the desired direction, mirroring the messaging of the course. Most consistent with the central theme of the course, the percentage of participants who reported cooking at home with mostly fresh foods 5–7 times in the prior week, increased from 63.4 % to 71.4 % (*p* < .0001). Similarly, when describing yesterday’s dinner, participants’ food choices reflected significant improvements that were aligned with the longitudinal health messaging in the course, including a significant increase in consumption of fresh vegetables (71.4 % to 77.3 %) and fresh fruits (28.4 % to 34.2 %) (*p*s < .0001). Comparatively little change was seen from pre- to post-course survey with regard to the consumption of foods that had neither been promoted nor discouraged in course videos (e.g., dairy).

**Table 2:** Pre- to post-survey changes in self-reported eating behaviors and meal composition (*N* = 7,422)

	Pre-survey		Post-survey		
	#	%	#	%	*P*-value
Prior Week Dinner					
# Days Eaten at Home					<.0001
0	83	1.1	140	1.9	
1–4	1160	15.7	870	11.7	
5–7	6169	83.2	6402	86.4	
# Days Child <18 ate at Home with a Family Member^a^					<.0001
0	78	2.1	53	1.4	
1–4	399	10.8	309	8.4	
5–7	3207	87.1	3322	90.2	
# Days Cooked at Home with Mostly Fresh Foods^b^					<.0001
0	396	5.5	396	5.5	
1–4	2220	31.0	1648	23.0	
5–7	4537	63.4	5109	71.4	
Yesterday’s Dinner					
Was Dinner Cooked at Home with Mostly Fresh Foods (% yes)	4902	66.1	5352	72.1	<.0001
*Did it include*					
Fresh Vegetables	5296	71.4	5738	77.3	<.0001
Fresh Fruit	2104	28.4	2525	34.2	<.0001
Poultry	2370	31.9	2463	33.2	.07
Fish	1141	15.4	1227	16.5	.03
Red Meat	2252	30.3	1979	26.7	<.0001
Vegetable Protein	1657	22.3	1919	25.9	<.0001
Dairy	3129	42.2	3162	42.6	.54
Starch	5331	71.8	5157	69.5	.0004
Baked Goods/Sweets	884	11.9	723	9.7	<.0001
Perceptions of Dinner					
*Enjoyment*					<.0001
Not/Somewhat Enjoyable	925	12.7	577	7.9	
Moderately Enjoyable	2351	32.2	1860	25.5	
Very/Extremely Enjoyable	4032	55.2	4871	66.7	
*Healthy*					<.0001
Not/Somewhat Healthy	1356	18.7	803	11.1	
Moderately Healthy	3033	41.9	2352	32.5	
Very/Extremely Healthy	2848	39.3	4082	56.4	

Note. Ns do not always add up to 7,422 due to missing responses. ^a^Includes only participants with a child < 18 years old (*N* = 3,915). ^b^Includes only participants who reported they ate at home at least once in the past week at each survey (*N* = 7153). Results based on McNemar-Bowker tests of symmetry

Finally, there was a significant increase in the percentage of participants who found yesterday’s dinner to be very or extremely enjoyable (55.2 % to 66.7 %; *p* < .0001) and those who felt that yesterday’s dinner was very or extremely healthy (39.3 % to 56.4 %; *p* < .0001).

Table 3 presents the changes in perceived ease or difficulty of making healthy food choices and preparing home cooked meals. All pre-post changes were in the hypothesized direction. For example, the percentage of participants who reported that it was easy or very easy to make healthy choices increased significantly across surveys, ranging from an increase of 5.7 % (get fresh foods to cook healthy meals) to 15.1 % (eat healthy) across surveys (*p*s < .0001). Mean overall perceived ease of making healthy food choices and preparing home cooked meals also increased significantly over time from 27.3 (SD = 5.7) to 29.1 (SD = 5.4) (*p* < .0001).

**Table 3:** Pre- to post-survey changes in perceived ease or difficulty of making healthy food choices and preparing home cooked meals (*N* = 7,422)

	Pre-survey		Post-survey		
	#	%	#	%	*P*-value
How Easy or Difficult Is It To:					
Avoid Processed Foods					<.0001
Easy/Very Easy	3300	45.7	4030	55.8	
Neutral	2043	28.3	1930	26.7	
Difficult/Very Difficult	1879	26.0	1262	17.5	
Get Fresh Foods to Cook Healthy Meals					<.0001
Easy/Very Easy	4814	66.6	5225	72.3	
Neutral	1637	22.6	1366	18.9	
Difficult/Very Difficult	778	10.8	638	8.8	
Decide What to Cook					<.0001
Easy/Very Easy	2442	33.9	3139	43.5	
Neutral	2047	28.4	2118	29.4	
Difficult/Very Difficult	2720	37.7	1952	27.1	
Cook Using Fresh Foods					<.0001
Easy/Very Easy	5006	69.4	5672	78.6	
Neutral	1436	19.9	1102	15.3	
Difficult/Very Difficult	776	10.8	444	6.2	
Eat the Right Amount of Food					<.0001
Easy/Very Easy	2726	37.8	3535	49.0	
Neutral	2128	29.5	1977	27.4	
Difficult/Very Difficult	2364	32.8	1706	23.6	
Get Time to Cook a Meal at Home					<.0001
Easy/Very Easy	2738	37.9	3376	46.8	
Neutral	2163	30.0	2213	30.6	
Difficult/Very Difficult	2320	32.1	1632	22.6	
Cook a Meal that you Enjoy					<.0001
Easy/Very Easy	4928	68.1	5678	78.5	
Neutral	1501	20.8	1114	15.4	
Difficult/Very Difficult	805	11.1	442	6.1	
Eat healthy					<.0001
Easy/Very Easy	3811	52.8	4904	67.9	
Neutral	2234	30.9	1730	24.0	
Difficult/Very Difficult	1175	16.3	586	8.1	
	Mean	SD	Mean	SD	*P*-value
Total Ease of Healthy Cooking	27.3	5.7	29.1	5.4	<.0001

Note. Ns do not always add up to 7,422 due to missing responses. Results based on McNemar-Bowker tests of symmetry and paired *t*-tests

Results from stratified analyses that tested for changes in key variables (i.e., consumption of vegetables at dinner, consumption of fruits at dinner, total perceived ease of healthy cooking) by demographic subgroups from the pre- to post-survey (data can be assessed by contacting author) indicated that while males, younger adults, those with less education, US residents, and those who perceived themselves to be overweight or obese, tended to consume fewer fruits and vegetables and had lower perceived ease of healthy cooking at baseline, each demographic subgroup reported significant improvements in their consumption of vegetables, consumption of fruits, and total perceived ease of cooking healthy foods (*p*’s < .05).

## Discussion

The current study reports on the first cooking and nutrition MOOC created for an international audience as a public health intervention to improve eating behaviors and meal composition of the participants. Course participants reported significant positive changes in eating behaviors and meal composition over time, including an increase in the reported frequency of cooking dinner at home, using mostly fresh ingredients, as well as significant increases in the perceived health and enjoyment of home-cooked meals.

Composed of short, easy to understand videos aimed at conveying basic nutrition education and basic cooking skills, this MOOC was primarily intended to be a public health education resource for a large, international audience. The pilot intervention topics (e.g., teaching cooking skills, promoting home cooking) were designed to be broadly generalizable to the adults from over 80 countries who participated in the course. Among the 7,422 participants who completed both pre and post course surveys, significant positive changes were seen in most behaviors related to the key health messages emphasized throughout the course, including cooking at home, using mostly fresh ingredients, eating family meals; and practicing dietary moderation. Of particular interest were the findings that younger adults, those with less education, US residents, and those who perceived themselves to be overweight or obese, tended to consume fewer fruits and vegetables at the outset of the course and had lower perceived ease of healthy cooking. However, each of these subgroups experienced significant improvements in their consumption of vegetables and fruits, and total perceived ease of cooking healthy foods, suggesting that these higher risk individuals may have benefitted from the course. Future studies that are able to tailor their messaging for specific sub-populations (e.g., country of origin) may have an even larger impact.

Past literature has shown that both face-to-face and online nutrition education can engage students and increase nutrition knowledge. Ha and Caine-Bish [4] delivered an interactive introductory nutrition course to college students, combining didactic nutrition education with activities such as diet and exercise self-tracking. Whole grain intake increased significantly among the 80 students who participated in the study. A registered dietitian at Vanderbilt University offered a nutrition MOOC without an instructional cooking component [36]. Feedback shared through discussion forums was overwhelmingly positive, although dietary changes pre-post course were not published. These studies underscore the potential for disseminated nutrition education to result in improved knowledge and eating behaviors, yet few studies have evaluated the efficacy of delivering cooking skills instruction and nutrition education via MOOCs.

### Strengths and future directions

The relatively large sample size in this study as well as the broad, international composition of the participant body, add to the potential impact of this analysis. Since 2014, the Stanford Child Nutrition and Cooking MOOC has been offered as an “on-demand” course on Coursera, allowing participants to enroll at any time and complete the course at their own pace. By October 2015, course enrollment for this MOOC had reached 228,635. Maintenance and course direction require a minimal time investment on the part of the instructor and the online platform staff once course content has been created, so the potential for sustainability of this educational resource is high.

### Limitations

There are several limitations to this analysis. First, only participants who completed both pre- and post-course surveys could be included; however, attrition analyses indicated that participants who completed both the pre- and post-surveys were generally similar to those who only completed the pre-survey on demographic characteristics. Because enrollment in MOOCs is free and easy, large differences are seen between enrollment rates and course participation rates. Researchers have observed a characteristic “funnel of participation” [35, 37] with only approximately half of enrollees active on the course site following their enrollment [37]. Documented average completion rates for MOOCs are variously reported as “less than 10 %” of enrolled students completing the course [38] or “generally between 10 and 20 %” [39]. The completion rate for the MOOC described here was higher than the reported averages, with 30 % of students enrolled earning a Statement of Accomplishment, but it has been suggested that completion rates do not accurately represent student engagement [37] as many students view video lectures without completing assessments. Assessing engagement has been identified as a major challenge in determining the effectiveness of MOOCs as public health education tools [37]. We also faced this challenge in our efforts to accurately assess engagement.

Second, participants’ eating behaviors were self-reported and the social desirability of positive changes may have influenced responses; however, the variability in changes seen across variables lends credibility. Similarly, participants who completed both surveys may have been more motivated and thus, more likely to improve eating behaviors, than those who failed to complete the course. Third, individual-level data on dose of exposure to course materials is not available and thus we cannot determine the depth of engagement for individual participants and how this related to changes in eating behaviors. Fourth, the majority of participants in this study were women, 20–49 years of age, who lived with a family and had at least some post-secondary education. Fewer participants were men, older adults, and those who live alone, suggesting opportunities for targeted outreach to these groups. Fifth, The Stanford Child Nutrition and Cooking MOOC was offered only in English. Subsequently, the course was translated into several world languages and is now being offered with the option of turning on subtitles in Korean, Spanish, Russian, Ukrainian, Dutch, Bulgarian and Croatian. Sixth, all survey measures were designed for this study by the team of medical and nutrition science experts in the Stanford School of Medicine for program evaluation. Demographic questions were adapted from U.S. national surveys (i.e., BRFSS, NHANES). Self-efficacy scales were adapted from previously evaluated nutrition interventions [40] and tailored to our intervention. Additional research is needed to validate these questions.

Finally, computer access and literacy is likely to be an ongoing barrier to participation in online courses, as evidenced by the clustering of higher education levels among the majority of course participants.

## Conclusions

The progressive substitution of highly processed foods with healthy home-cooked alternatives is a potentially powerful behavioral shift that aligns well with existing efforts to improve eating behaviors and meal composition. Based on the findings of this study, a valuable approach to supporting this shift, may be found in the creation of accessible, integrated nutrition education and cooking instruction. The use of the MOOC format for delivering public health education of this kind allows participants to overcome logistical barriers to more traditional forms of education. With further evaluation, MOOCs have the potential to surpass traditional didactics as effective and far-reaching public health tools. More research is needed to determine how MOOC content can be tailored to maximize accessibility and positively impact the behaviors of people who may not otherwise have access to basic health education.

### Endnote

^1^Note: The data analysis for this paper was generated using SAS software. Copyright, SAS Inc. SAS and all other SAS Institute product or service names are registered trademarks of SAS institute Inc., Cary, NC, USA.

## Abbreviation

MOOC: Massive open online course
